# Advanced Technologies to Target Cardiac Cell Fate Plasticity for Heart Regeneration

**DOI:** 10.3390/ijms22179517

**Published:** 2021-09-01

**Authors:** Gianluca Testa, Giorgia Di Benedetto, Fabiana Passaro

**Affiliations:** 1 Department of Medicine and Health Sciences “V. Tiberio”, University of Molise, 86100 Campobasso, Italy; gianluca.testa@unimol.it; 2 Interdepartmental Center for Nanotechnology Research—NanoBem, University of Molise, 86100 Campobasso, Italy; 3 Department of Molecular Medicine and Medical Biotechnology, Federico II University, 80138 Naples, Italy; giorgia.dibenedetto@unina.it

**Keywords:** iPSC, direct cardiac reprogramming, heart failure, nanoparticle, cardiac targeting

## Abstract

The adult human heart can only adapt to heart diseases by starting a myocardial remodeling process to compensate for the loss of functional cardiomyocytes, which ultimately develop into heart failure. In recent decades, the evolution of new strategies to regenerate the injured myocardium based on cellular reprogramming represents a revolutionary new paradigm for cardiac repair by targeting some key signaling molecules governing cardiac cell fate plasticity. While the indirect reprogramming routes require an in vitro engineered 3D tissue to be transplanted in vivo, the direct cardiac reprogramming would allow the administration of reprogramming factors directly in situ, thus holding great potential as in vivo treatment for clinical applications. In this framework, cellular reprogramming in partnership with nanotechnologies and bioengineering will offer new perspectives in the field of cardiovascular research for disease modeling, drug screening, and tissue engineering applications. In this review, we will summarize the recent progress in developing innovative therapeutic strategies based on manipulating cardiac cell fate plasticity in combination with bioengineering and nanotechnology-based approaches for targeting the failing heart.

## 1. Introduction

Lacking any regenerative potential [1], the adult human heart can only adapt to heart diseases by starting a myocardial remodeling process to compensate for the loss of functional cardiomyocytes, which ultimately develops into heart failure [2]. Heart failure (HF) represents the outcome of several disorders, either cardiovascular or systemic and non-cardiac conditions, in which the activation of neurohormonal, inflammatory, and mechanical pathways lead to the development of cardiac hypertrophy and fibrosis, with consequent reduction of cardiac output [2,3]. In recent decades, with the increase in the elderly population, the incidence and prevalence of HF have been increasing, with a mortality rate of over 35% after acute decompensation [3]. 

In healthy adult hearts, cardiomyocytes account for approximately 30% of all cells, whereas the remaining cell types are non-cardiomyocytes, such as immune cells, vascular endothelial cells, and fibroblasts, with the latter comprising ~11% of the total heart resident cell population [4]. Since cardiomyocytes are terminally differentiated cells with no potential for self-renewal, they become necrotic and die upon injury. This condition results in scar formation by deposition of fibrotic tissue, which ultimately reduces the cardiac systolic function [5]. This fibrotic process is guided by activated cardiac fibroblasts (CFs) and can either be induced by an acute event, such as myocardial infarction (MI), to replace the massive loss of cardiomyocyte (the so-called reparative fibrosis), or by chronic, non-ischemic, injuries such as diabetes, hypertension, or obesity, all characterized by a systemic low-grade chronic inflammation leading to structural and/or functional abnormalities entailing the deposition of collagen and formation of fibrosis (reactive fibrosis) [5].

Mounting evidence suggests that cardiac regeneration can be mainly achieved by reprogramming non-cardiomyocytes into cardiomyocytes, differentiating pluripotent stem cells into cardiomyocytes, and re-activating the proliferation of pre-existing cardiomyocytes [6]. These approaches are based on the targeting of some key signaling molecules governing cardiac cell fate plasticity, among which there are a series of cell-cycle regulators, transcription and growth factors, non-coding RNAs, and other factors that have been shown to be involved in post-injury heart repair, and that may be used to develop innovative cardiac regenerative drugs for clinical applications.

Possible therapeutic interventions would greatly benefit from a cell-specific targeting to foster the shift from the systemic to the cellular setting, and in this context, the application of nanotechnologies for tissue engineering and drug delivery holds great potentiality. This has inspired the development of new treatment strategies aimed at the identification of crucial players involved in cell fate plasticity and their specific nano-targeting for cardiac regeneration [5,7].

Since the latest findings in inducing cardiomyocyte cell cycle re-entry have been already extensively reviewed elsewhere in this special issue [8,9], here we will summarize the recent progress in the development of innovative therapeutic strategies based on manipulation of cardiac cell fate plasticity in combination with bioengineering and nanotechnology-based approaches for targeting the failing heart.

## 2. Repairing the Failing Heart with Cellular Reprogramming 

Since the discovery of cellular reprogramming and induced Pluripotent Stem Cells (iPSCs) generation in 2007 [10], novel possible therapeutic approaches involving the replacement of the damaged myocardium with new cardiomyocytes converted from patient’s own cells have been proposed, laying the foundations for cardiac regenerative medicine [11]. 

Human iPSCs (hiPSCs) are similar to human embryonic stem cells (hESCs) in morphology, gene expression profile, epigenetic state, and differentiation potential, as they can differentiate into cells of all three embryonic germ layers, including cardiomyocytes [11]. Patient-derived hiPSCs carry the genome of their cell of origin, representing a powerful cell-based system for modeling human cardiovascular disease (CDSs), for genetic investigations and drug screening [12,13,14,15]. Moreover, patient-derived hiPSCs could provide a potentially unlimited source of cardiac precursor cells allowing the generation of cardiac cell lineages and bypassing the ethical challenges accompanying the use of hESCs for personalized therapy [12].

Although hiPSCs undoubtedly represent an exceptional technology for in vitro diseases modeling, their clinical translation is still considerably hampered by the lack of scalable differentiation protocols and the potential tumorigenic activity. Conversely, the possibility to directly reprogram somatic cells into induced cardiomyocytes (iCMs) without passing through a pluripotent state, the so-called Direct Cardiac Reprogramming (DCR), has become a promising strategy to produce functional cells in vivo for therapeutic purposes, and significant progress has been made over the last decade in identifying the optimal DCR protocol as well as the underlying molecular pathways [16]. CFs are particularly useful due to their phenotypic plasticity and because they are present in large numbers in heart tissue [17]. Indeed, most of the strategies of DCR reviewed in this paper involve CFs reprogramming into iCM. 

Regardless of the reprogramming strategy, the acquisition of a new phenotype passes through the modification of gene regulatory networks of the resident cell type to fit the characteristics of the incoming target cell, in terms of cell behavior, proliferation, and metabolic rate [18]. These same changes can also be associated with some pathological cellular states, like tumorigenesis [19,20]. Therefore, adequate control of somatic cell plasticity is mandatory before reprogramming could be considered a useful tool for cardiac cell therapy and tissue engineering.

## 3. Challenges and Opportunities of hiPSC-Derived Cardiomyocytes 

iPSCs are generated by forcing the expression of specific transcription factors including OCT4, SOX2, c-MYC, and KLF4 [10] in combination with LIN28 and NANOG [21] into adult somatic cells. Different human adult cells have been successfully reprogrammed into hiPSCs, like dermal fibroblasts [21], CFs [22], endothelial cells [23], keratinocytes [24], hair follicle cells [25], and peripheral blood cells [26]. After the validation of their pluripotent condition [27], hiPSCs can be differentiated into mesodermal derivatives to allow the generation of cardiac cell lineages, including cardiomyocytes [28]. 

Forerunner studies on animal models for MI have already demonstrated that transplantation of iPSC-derived CMs (iPSC-CMs) successfully improved the cardiac phenotype and attenuated cardiac remodeling [29,30,31,32,33], although some concerns on engraftment efficiency and teratogenicity still need to be overcome. At the same time, hiPSCs technology offers the best system for understanding the genetic basis and molecular pathways involved in human CVDs, for the creation of patient-specific disease models to test the pathological relevance of gene mutations, for drug testing, discovery and development [13,14,34]. 

### 3.1. Strategies for hiPSC Differentiation into Mature Cardiomyocytes 

Two principal strategies have been used to promote hiPSC differentiation into cardiomyocytes. Initial protocols were based on the generation of single iPSCs suspension cultures which spontaneously aggregate and form embryoid bodies (EBs), tridimensional (3D) structures in which cells differentiated into the three germ layers mimicking embryogenesis [35]. Following EB formation, cells were moved into culture plates for cell adhesion to promote the acquisition of CM properties [28,36]. Later, modifications to the original EB-based protocol have been proposed to increase the efficiency of differentiation. Such improvements employed combinations of cytokines and growth factors [37,38] to imitate the signaling pathways involved in embryonic cardiovascular development and CM maturation.

Subsequently, simplified procedures based on monolayer cell cultures (2D) in place of EB formations have been established. These differentiation protocols enabled the derivation of cardiac progenitor cells (CPCs) from hiPSCs, followed by a second differentiation step to promote the maturation of different cardiovascular cell subtypes [39,40]. The optimization of cell culture conditions allowed to obtain hiPSC-CMs with >90% purity [41]. Interestingly, the purity of hiPSC-CMs derived from reprogrammed CFs exceeded 92%, showing the higher rate among iPSCs derived from different somatic cell types [22], probably for the presence of some epigenetic features retained from their tissue of origin [42]. Furthermore, a comparison of hiPSC-CMs derived from cardiac-derived mesenchymal progenitor cells, bone marrow-derived mesenchymal stem cells, and human dermal fibroblasts from the same patient showed that cardiac somatic cells presented the best rate for CMs differentiation due to upregulated cardiac genes [43], confirming that the origin of the starter somatic cells is a determinant of iPSC-CMs maturation. 

Unfortunately, differentiation approaches reported so far have limitations in generating immature CMs resembling fetal cells in their function, morphology, and electrophysiology [44]. Additionally, tumors can form during in vitro culture of iPSCs, increasing the malignant risks for in vivo applications [45]. All of this constitutes an important obstacle to using hiPSC-CMs to treat CVDs that must be addressed [11].

Several approaches were attempted to overcome the issue of hiPSC-CMs immaturity, such as the long-term culture [46,47], the use of hormones [48], metabolic substrates [49,50] and microRNAs [51]. Recently, it has been observed that inhibition of the mechanistic target of the rapamycin (mTOR) pathway enhances hiPSC-CMs maturation by shifting cells to a quiescent state, which enhances cardiomyocyte maturity. [52]. 

Among the methods developed to increase the maturation of iPSC-CMs, the modulation of extracellular matrix (ECM) surrounding hiPSC-CMs provided great results. One example is the so-called “matrix sandwich” method, in which confluent iPSCs are covered with a matrix of Matrigel mixed with a combination of specific growth factors and cytokines to induce cardiac differentiation [53], as well as the use of synthetic culture matrices engineered from combinatorial polymers [54]. Even better results were obtained by placing hiPSCs under the influence of physiological cyclic pulsatile hemodynamic forces within a microfluidic system [55,56]. Indeed, compared to static cultures, hiPSC-CMs cultured in microfluidic systems showed increased cell size, alignment, contractility, sarcomere length and appeared more rod-shaped [56]. Furthermore, considering that mature iPSC-CMs have a higher oxygen consumption rate with increased mitochondrial maturity with respect to the immature counterpart, it has been designed a metabolic flow technology to enable the large-scale purification of mature iPSC-CMs, through glucose depletion and lactate supplementation [57]. 

**Figure 1:**
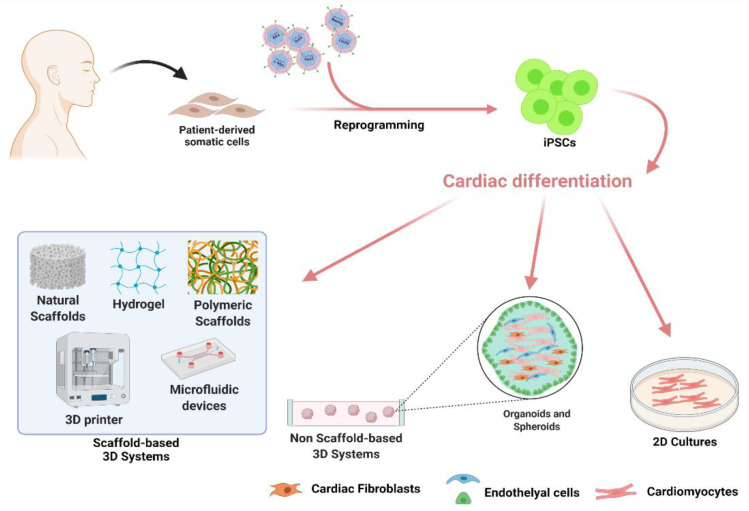
Schematic representation of induced Pluripotent Stem Cells (iPSCs)-derived cardiac cell generation and their possible use in translational medicine. The figure is created with BioRender (https://biorender.com/, accessed on 30 July 2021).

Despite a large effort for protocol improvements, the main limitation of 2D cultures remains the inability to mimic the in vivo heart complexity, either in terms of spatial architecture or of multi-cellular interactions, which have been demonstrated to be crucial for CMs maturation in vivo [58,59,60]. To address this problem, research started to focus on the development of 3D culture models re-creating in vitro a reliable 3D tissue, which would be certainly more effective to closely mimic the in vivo structure, microenvironment, cell–cell and cell–ECM interactions. In this framework, the hiPSCs technology in partnership with nanotechnologies and bioengineering [61] offers new perspectives in the field of cardiovascular research for disease modeling, drug screening, and tissue engineering applications (Figure 1).

### 3.2. Non-Scaffold-Based 3D Systems for In Vitro Modelling of Cardiovascular Diseases 

Non-scaffold-based 3D systems are typically hiPSC-derived spheroids [62] and hiPSC-derived organoids [63]. Cardiac spheroids are established in a self-assembly process by co-culturing in suspension hiPSC-CMs with CFs and cardiac Endothelial Cells (ECs) in order to closely recapitulate the native microenvironment of the myocardium [64,65]. Self-aggregation of cells in these 3D, sphere-shaped structures is promoted by low-adhesion culture conditions, allowing suspended cells to attach to each other. However, spheroids cannot regenerate and are unable to fully replicate the intricate heart tissue microenvironment. While spheroids develop primarily via cell-to-cell adhesion, internal developmental processes drive organoid formation. Cardiac organoids, in fact, develop from a single stem cell or iPSC capable of self-renewing and differentiating into multiple lineages in vitro by manipulating the culture environment through integration with organ-specific growth factor cocktails. These growth factors shape the stem cell niche environment during physiological tissue self-renewal or damage repair. This results in a multicellular 3D structure made up of different cell lineages that reflect the important structural and functional properties of organs, recapitulating organ-like tissue architecture and cellular composition [66,67]. 

The presence of different cardiac cell types offers the opportunity to model those interactions which may be decisive to establish a pathological phenotype [14], making non-scaffold-based 3D systems either valuable tools for modeling CVDs in a patient-specific fashion, allowing the simultaneous study of a large variety of phenotypes, or a robust technology applicable in drug screening and development for personalized medicine. 

Patient-specific hiPSC-based cardiac spheroids and organoids recreate the 3D cardiac and vascular networks in which external environmental stimuli influence cardiac tissue development and maintenance. The multiple cell cultures of cardiac microvascular ECs together with human CFs in the formation of cardiac spheroids also resulted in an increased maturation of hiPSC-CMs, as demonstrated by a better tissue organization and synchronous beating of hiPSC-CMs, and formation of blood capillary-like networks [68]. With the aim to induce the blood vessel organoid, Wimmer et al. developed a two-step protocol by first differentiating hiPSCs in suspension into the mesodermal lineage before inducing the EC differentiation. When implanted in the mouse kidney, the blood vessel organoids displayed morphological and functional similarities with native human blood vessels [69]. 

A recent characterization of hiPSC-CMs in tri-culture revealed improved microvasculature and increased contraction rate in 3D microtissue spheroids when compared with control 3D spheroids [70].

3D human cardiac organoids have been used for the screening of a panel of environmental toxins, assessing organoid beating activity and viability [71]. In another study, a high-throughput human cardiac organoid system has been used to screen more than 100 small molecules with presumed cardiac pro-regenerative potential [72], allowing the identification of two very promising pro-proliferative effectors.

However, the self-assembly process is a random procedure that results in heterogenous organoids in terms of cell composition, size, and shape, which limit the application of this method to regenerative medicine [73].

### 3.3. iPSC-Derived Cardiac Cells and Scaffold-Based 3D Systems for Tissue Engineering Applications

Scaffold-based 3D systems rely on combining cells and biocompatible scaffolds to recreate a functionally native tissue, recapitulating the exact cellular composition and ECM structure. The final purpose of such tissue engineering (TE) methodologies is of high clinical relevance, as they aim at replacing the diseased heart tissue [61,74]. 

Scaffolds are responsible for the structural support of the seeded cells, while also affecting functional aspects of cell behavior (proliferation rate, survival, differentiation) [61]. Indeed, cell culture in 3D scaffolds promotes the maturation of hiPSC-CMs inducing T-tubules formation, normally absent in 2D cultures, and improving the structural and functional maturation of CMs [74]. Thus, the molecular composition of the scaffold is a crucial feature and TE technologies used either synthetic (e.g., lactide and glycolide copolymer, polylactide or polyglycolide, polycaprolactone) or natural (e.g., collagen, silk fibroin, cellulose, hyaluronic acid, chitosan, decellularized ECM) polymers in combination with hiPSCs derived cells to provide the best tool for specific applications [61].

The excellent biological activity and the fact that many of them are naturally present in the human body make the group of natural scaffolds the most popular among the backbone used to date. However, their shortcomings are their low mechanical strength, the potential to induce immune responses, and batch-to-batch variability [75]. Synthetic hydrogel polymers, on the other hand, exhibit a slightly lower degree of biocompatibility than purified natural hydrogels. Still, they have received a lot of attention due to their powerful mechanical properties, ease of control, low immunogenicity, and no batch shifting issues. in cardiovascular regeneration [75].

A valuable 3D heart model has been generated by combining hiPSC-derived CPCs and mouse decellularized heart matrix [76]. This study reported the ability of ECM to promote hiPSC-CMs differentiation and proliferation, generating an engineered heart tissue with the typical myocardium structure and the expected electrophysiological characteristics [76]. 

Another approach relies on loading the desired cells into polymers to generate sheets of spontaneously beating CMs, the so-called “heart-on-chip” tissue, mimicking the human myocardium [77]. This method has been applied by seeding hiPSC-CMs from patients with Barth syndrome-related cardiomyopathy onto thin elastomers micropatterned with fibronectin lines, generating self-organized laminar myocardium that featured aligned sarcomeres [78]. 

Cell-based cardiac patches have been designed to increase the survival ratio of the embedded cells and to ensure cellular retention. Human cardiac muscle patches have been generated by suspending hiPSC-CMs, smooth muscle cells, and ECs in a fibrin scaffold. Transplantation of this patch in a porcine model of MI resulted in significantly reduced infarct size and improved cardiac function associated with a reduction in left ventricular wall stress [79]. Human-engineered heart tissue patches containing iPSC-CMs and transplanted into a guinea pig cardiac injury model resulted in a partial remuscularization of the diseased heart and improved left ventricular function in a dose-dependent manner [80]. In the same study, human-scale patches were successfully transplanted in pigs as a proof-of-principle study [80].

Very recently, Zhu et al. presented an innovative injectable patch that will revolutionize the delivery of cardiac patches, which usually needs a traumatic open-chest surgery [81]. They developed and tested in rodent models of MI a method to utilize the pericardial cavity for in situ cardiac patch formation after intrapericardial injection of biocompatible hydrogels containing iPSC-derived CPCs or mesenchymal stem cells-derived exosomes. After injection, the hydrogels formed a cardiac patch-like structure in the pericardial cavity, mitigating immune response and increasing the cardiac retention of the therapeutics. Moreover, they obtained a robust cardiovascular repair that improved cardiac functions post-MI [81]. 

In recent years, 3D bio-printing has also been used to recreate functional hiPSC-based cardiac tissues [82,83,84]. The microenvironment of printed tissue accurately resembles native conditions, which promotes complex tissue formation in vitro. Vascularized patches were successfully printed using a biopsy of fat tissue [85]. In brief, part of the sample was used to extract the cells to be reprogrammed into hiPSC, which have been subsequently differentiated into hiPSC-CMs and hiPSC-ECs, whereas the remaining part was decellularized and processed to generate a patient-personalized hydrogel, which served as a bio-ink for 3D printing. Then, anatomical data obtained from computerized tomography of a patient’s heart were used to design patch dimensions and geometry of blood vessels to obtain a personalized scaffold [85]. Finally, differentiated cells were incorporated into the personalized hydrogel to form a bio-ink for the parenchymal cardiac tissue and blood vessels matching the immunological, cellular, biochemical, and anatomical properties of the cell donor [85].

The proper modeling of the myocardium should also include electrical [77] and mechanical [86] stimuli, such as those derived from hydrostatic pressure. In 2013, Nunes et al. proposed the innovative platform “Biowires”, combining a 3D system with electrical stimulation inducing highly organized cardiac structure and maturation of hiPSC-derived cardiac tissues [87]. Later, the platform was improved by using long-term electrical stimulation. This “Biowires II” platform generated a 3D human-based cardiac tissue model displaying adult-like properties [88]. 

The combination of both electric and mechanical stimulation was even more effective in the creation of advanced hiPSC-derived cardiac microtissues [89]. Moreover, mechanical loading promoted hiPSC-CMs maturation in terms of sarcomere length, improved calcium handling and increased CMs marker gene expression [90,91]. 

One of the main challenges in the use of polymeric scaffolds is that the majority of polymeric materials used for tissue engineering are electrically insulated at biologically relevant frequencies [92] and thus do not conduct electrical signals that are critical to cardiac tissue function. To overcome this problem, in order to improve the electrical properties of polymeric materials, conductive nanoparticles, such as carbon nanotubes (CNTs) have been incorporated into different scaffold systems [93]. Such nanoengineered hybrid systems were revealed to be supportive of long-term CMs viability, maturation, and functionality compared with traditional polymeric scaffold systems without CNTs [94].

The combination of 3D scaffolds and hiPSC technology might be useful to establish new approaches for the treatment of a variety of cardiovascular-related defects, such as to produce vessels and/or valvular constructs [95] or to manufacture vessel substitutes, which presented similar mechanical resistance as clinically used prosthesis [96,97]. 

These pioneering studies have yielded promising preclinical results that must be implemented before hiPSC-derived cardiac cells can be projected towards a safe and effective translation in the clinical setting. Major concerns are related to the possible chromosomal aberrations [98], which can be inherited from parental cells or result from cell reprogramming or extended culture periods, as well as the potential long-term deleterious effects of the implanted engineered tissue, the lack of heterogeneous cell population after differentiation into CMs, and the inadequate maturity of CMs. Therefore, a significant challenge now is developing standardized protocols for reproducible production of high-quality hiPSC-CMs, to be used for studying CVDs and possible clinical applications.

## 4. Direct Cardiac Reprogramming of Cardiac Fibroblasts into Cardiomyocytes

After MI, CFs are activated and recruited to the injured site to form scar tissue replacing the necrotic heart muscle. Therefore, reprogramming these abundant cell populations into functional CMs would be an ideal strategy for heart repair in response to ischemic injury [7]. The process of converting somatic cells from one lineage to another without transitioning through an intermediate pluripotent state is known as direct reprogramming [16] and, compared to hiPSCs reprogramming, enables a faster and more efficient conversion of cells in situ without the need for ex vivo cell expansion and transplantation.

Direct reprogramming has been achieved for several human and mouse cell types by the forced expression of transcription factors and/or non-coding RNAs or through the delivery of small molecules modulating crucial pathways [16]. Nonetheless, in vivo studies performed in mice have highlighted a number of challenges that remain to be overcome before this approach could be used in the human clinic, like the low efficiency of conversion, the immaturity, at least in vitro, of reprogrammed cells, the absence of safe delivery methods, and the inability to precisely direct differentiation towards the desired cell subtype [7,16]. Generation of multipotent iCPCs instead of iCMs may offer some advantages for heart regeneration as they have the potential to differentiate into different cardiac cell lineages, retaining a certain ability to proliferate [99].

### 4.1. Strategies for In Vitro Direct Cardiac Reprogramming

Since the first report of the conversion of mouse embryonic fibroblasts (MEFs) into myoblasts by forced expression of the myoblast determination protein 1 (MYOD) [100], several studies elucidating the molecular mechanisms of DCR have led to significant improvement of reprogramming efficiency by refining the cocktails of reprogramming factors, in parallel with the development of innovative methods for reprogramming factor delivery [101].

Reprogramming requires the inhibition of the fibroblast signatures with the parallel enhancement of cardiac fate, which involves a drastic chromatin remodeling occurring in the starting fibroblast to overcome existing epigenetic barriers and to acquire the CM-like chromatin pattern [16]. The success of such changes greatly influences the efficiency and outcome of reprogramming.

Lineage-specific pioneer transcription factors (TFs), that bind and open closed chromatin to enable the binding of other canonical TFs [102], are typically included in most reprogramming cocktail combinations. GATA binding protein 4 (GATA4) is the pioneer in both human and mouse cardiac reprogramming [103] and is necessary for DCR mediated by forced expression of TFs [104,105]. GATA4 co-operates with other TFs to synergistically activate cardiogenic loci. Among the binding partners, Myocyte Enhancer Factor 2C (MEF2C) and T-Box Transcription Factor 5 (TBX5) are required to activate a cardiomyocyte gene program in fibroblasts [106]. The combination of GATA4, MEF2C, and TBX5 (referred to as GMT) was the first and more effective in inducing mouse DCR [104], followed by several attempts based on the addition of other TFs to the GMT core, like Heart And Neural Crest Derivatives Expressed 2 (HAND2) alone (referred to as GHMT) [105], with AKT Serine/Threonine Kinase 1 (AKT1) (referred to as AGHMT) [107], or with NK2 Homeobox 5 (NKX2.5) (referred to as NGHMT) [108]. The addition of AKT1 to the GHMT protocol increased spontaneous beating in reprogrammed iCMs and produced cells that were responsive to β-adrenoreceptor modulation, suggesting the acquisition of a more mature phenotype. Insulin-like Growth Factor 1 (IGF1) and Phosphoinositol 3-Kinase (PI3K) worked upstream of AKT1, whereas the mTOR complex 1 (mTORC1) and Forkhead box o3 (FOXO3A) acted downstream of AKT1 to influence CFs reprogramming into iCMs. Moreover, AKT1 overexpression was not associated with altered proliferation or apoptosis [107]. The role of mTORC1, however, is still controversial as Wang et al. observed enhanced reprogramming efficiency following rapamycin treatment, which they attributed to increased autophagy due to mTORC1 inhibition [109], in contrast to the impaired reprogramming observed by Zhou et al. upon mTORC1 inhibition [107]. 

These studies demonstrated that reprogramming factors are not equally important, and their stoichiometry is crucial for the good progress of DCR [110]. Despite lacking pioneering ability, MEF2C plays a key role in the initial up-regulation of cardiac gene expression and late maturation of iCMs, both in vitro and in vivo [110,111,112].

Modification of the GMT combination by the addition of other TFs proved to be more effective also for human DCR, like the overexpression of GMT with Mesoderm Posterior BHLH Transcription Factor 1 (MESP1) and Myocardin (MYOCD) [113] or the combination of GMT factors plus MESP1, MYOCD, Estrogen-Related Receptor Gamma (ESRRG), and Zinc Finger Protein, FOG Family Member 2 (ZFPM2) [114]. 

In the last two decades, different studies made important advances in identifying microRNAs (miRs), a group of small non-coding RNAs, as pivotal players in regulation of cell fate plasticity [115]. miRs have emerged as functionally critical regulatory molecules in DCR, guiding processes like de novo DNA methylation, progression of the cell cycle, and cell fate decision. The use of miRs driving DCR has been explored as an alternative to TFs overexpression. A cocktail of miR-1, miR-133, miR-208, and miR-499, known as “miR combo”, induced the expression of cardiac marker genes up to 7.7% in mouse neonatal CFs [116] and upregulated the expression of endogenous GHMT reprogramming factors [117]. Combination of miRs with TFs also showed an increasing in the reprogramming efficiency, in both MEFs and human CFs, like the addition of miR-133 to GMT-MYOCD-MESP1 cocktail, sufficient to reprogram human CFs into iCMs [118], the addition of miR-1 and miR-133 to GHMT [119] or to GMT-MYOCD-NKX2.5 [120] which dramatically increased the percentage of spontaneously contracting iCM from human dermal fibroblasts up to 12%, with a significant upregulation of cardiac gene signatures and concomitant repression of pro-fibrotic genes. Additionally, they reported that the addition of Janus Kinase 1 (JAK1) and Glycogen Synthase Kinase 3 beta (GSK3β) inhibitors significantly enhanced DCR efficiency [120]. 

Changes in chromatin accessibility, which may decrease, increase or be transiently reconfigured, mainly occur in regions distal to the transcription start sites of specific loci [103] and are reflected in alterations of the DNA methylation pattern and histone modification marks [113]. A global reconfiguration of DNA methylation landscape, which normally occurs upon switching from a cell fate to another [121,122], was observed during conversion to iCMs at promoters of genes that define the cardiac lineage, such as Natriuretic Peptide A (NPPA) and Myosin Heavy Chain 6 (MYH6), which became demethylated soon after GMT induction [123]. In parallel, marks associated with transcriptional repression, including trimethylation of Lysine 27 on Histone H3 (H3K27me3), which are often found within cardiac gene promoters and enhancers in fibroblasts, are removed and replaced by marks associated with transcriptional activation including H3K27ac, and trimethylation of Histone H3 Lysine 4 (H3K4me3—active promoters) upon transdifferentiation into iCMs [104,114,117,123,124,125]. In contrast, fibrotic genes accumulate H3K27me3 as reprogramming progresses [123,126]. 

Increased H3K4me3 levels at cardiac genes are also observed upon the knockdown of the Polycomb Repressive Complex 1 (PRC1) component B cell-specific Moloney murine leukemia virus Integration site 1 (BMI1), whose downregulation facilitates the removal of the transcriptionally repressive mark Histone H2A Lysine 119 ubiquitination (H2AK119ub), increasing the reprogramming efficiency [114,127,128]. As H3K27me3 is deposited by Enhancer of Zeste Homolog 1 and 2 (EZH1, EZH2) methyltransferases, their inhibition also promotes DCR [129], as well as the upregulation of lysine demethylases 6A and 6B (KDM6A and KDM6B), as by the overexpression of miR-1, miR-133, miR-208, and miR-499, which upregulated the expression of KDM6A in neonatal CFs by downregulation of EZH2 gene expression [117]. 

Knockdown of EZH2 also significantly increased human iCM reprogramming efficiency, leading to cardiac gene activation and repression of collagen and extracellular matrix genes. Furthermore, EZH2 inhibitors targeting its catalytic activity also promote human iCM reprogramming, suggesting that EZH2 may restrain cardiac conversion through H3K27me3-mediated gene repression [130]. 

Very recently, Garry et al. demonstrated that overexpression of the histone reader PHD Finger Protein 7 (PHF7) in mouse tail-tip fibroblasts (TTFs) generated a 10-fold increase of iCM with respect to AGHMT alone [131]. PHF7 with MEF2C and TBX5 alone induced the reprogramming of TTFs to iCMs in the absence of GATA4 by binding to multivalent cardiac super-enhancers through the recognition of H3K4me2/3 marks and increasing the chromatin accessibility and the binding of MEF2C and TBX5 at these sites [131]. The changes in chromatin accessibility were also due to the interaction of PHF7 with the cardiac-specific SWI/SNF subunit Smarcd3/BAF60C. Additionally, PHF7 was found to enhance reprogramming of adult human CFs, inducing a ~threefold increase in reprogramming above the MYOCD-containing human reprogramming cocktail alone [131].

### 4.2. Chemical Modulation of Signaling Pathways Governing Direct Cardiac Reprogramming 

**Figure 2:**
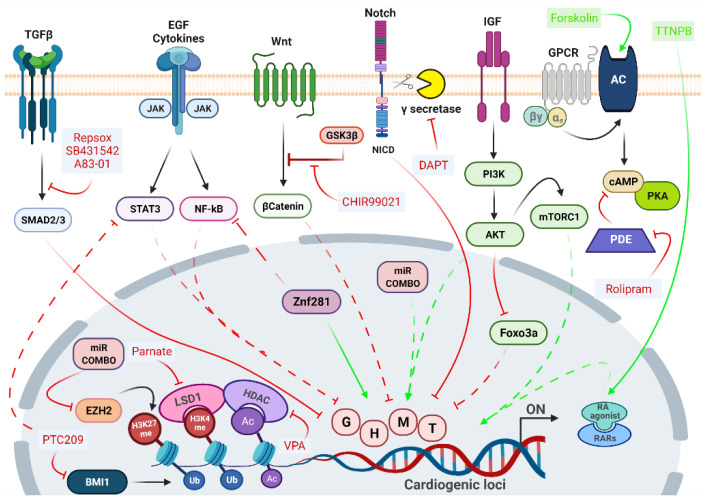
Molecular mechanisms involved in DCR. Green arrows indicate pathways that enhance reprogramming, while green text indicates molecules that activate the relative target. Red arrows indicate pathways that negatively regulate reprogramming, while red text indicate molecules that inhibit the relative target. Solid lines indicate direct interactions. Dashed lines indicate indirect interactions. Adenylyl Cyclase (AC); Notch Intracellular Domain (NICD); Retinoic Acid Receptor (RAR); miR-1, miR-208, miR-133, miR499 (miR Combo); Gata4 (G); Hand2 (H); Mef2c (M); Tbx5 (T); ubiquitylation on Lys119 (Ub); histone H3 Lys4 methylation (H3K4me); histone H3 Lys27 methylation (H3K27me); histone 3 Lys 27 acetylation (Ac). The figure is created with BioRender.

The epigenetic repatterning and the consequent remodeling of gene regulatory networks governing cell fate switches depend on the coordinated action of different signaling pathways, whose manipulation may dramatically enhance DCR efficiency. Several approaches in DCR adopted the addition of small molecules inhibiting fibroblast signatures and enhancing cardiac fate. The main signaling pathways and their targeting molecules are summarized in Figure 2.

The Transforming Growth Factor-β (TGF- β) pathway is one of the main signaling pathways active in fibroblasts, participating in the cell regulatory network through synergistic and antagonistic interactions with many other signaling routes [5,132]. The superfamily includes the TGFβ ligands, activins, nodal, Growth Differentiation Factors (GDFs) and Bone Morphogenetic Proteins (BMPs), which signal through specific transmembrane receptors [133]. The selective inhibition of TGF-β receptor prevents SMAD Family Members Smad2/Smad3 phosphorylation and the subsequent initiation of downstream signaling [133], which triggers the decrease of fibroblast gene expression programs by facilitating the Mesenchymal-to-Epithelial (MET) transition, and the suppression of profibrotic signals [124,134,135,136]. Small molecule inhibitors of the TGFβ pathway, like SB431542, RepSox, and A83-01 are frequently used for the direct conversion of fibroblasts into various cell types. Indeed, the addition of SB431542 to the GHMT/NKX2.5 cocktail produced a fivefold increase in iCMs generation [136], as well as the overexpression of GHMT plus miR-1 and miR-133 along with A83-01 efficiently promotes reprogramming [125]. As the fibrotic process requires the early activation of TGF-β signaling, followed by the later activation of Rho-associated kinase (ROCK) pathway, the addition of the ROCK inhibitor Y-27632 also inhibited the expression of pro-fibrotic markers, although less efficiently than A83-01 [134].

The negative regulation of JAK/STAT and NOTCH pathways was shown to improve DCR efficiency [107,114,120,134,135,137]. The inhibition of JAK/STAT enhanced the percentage of reprogrammed CFs following miR-1, miR-133, miR-208, and miR-499 delivery [116]. Inhibition of both Epidermal growth factor (EGF) and JAK2 signaling augmented reprogramming in fibroblasts by AGHMT factors [106]. The inhibition of γ-secretase by the DAPT compound, which impairs NOTCH maturation avoiding the nuclear translocation of its intracellular domain (NICD), in both GHMT- and AGHMT-reprogrammed fibroblasts, increased the percentage of cells expressing late cardiac marker genes such as cardiac Troponin T (cTnT) and α-actinin [137]. Moreover, Abad et al. also demonstrated that the inhibition of NICD nuclear translocation boosted the efficiency of DCR increasing MEF2C binding to cardiogenic loci [137]. 

These reports indicate that a prerequisite for reprogramming is the overcoming of possible related barriers, such as fibrosis with the associated gene network, highly expressed in postnatal and adult fibroblasts compared with embryonic fibroblasts. Considering the relationship between fibrosis and inflammation, we can assume that anti-inflammation may represent an additional potential target for lineage conversions, especially from a clinical translation perspective [138]. Indeed, very recent evidence indicates that the inhibition of pro-inflammatory pathways, like cyclooxygenase-2, Prostaglandin E2/prostaglandin E receptor 4, cyclic AMP/protein kinase A, interleukin 1β or IL6/Stat3 ameliorates DCR efficiency avoiding inflammation and fibroblast gene program [139]. We have also contributed to the field, demonstrating that in CFs the inhibition of Bmi1 expression by PTC-209 compound enhances DCR by repressing two major pathways related to inflammation, such as JAK/STAT3 and Mitogen-Activated Protein Kinase (MAPK)/ Extracellular signal-Regulated protein Kinase (ERK1-2) [128].

Together with the repression of the fibroblast gene network, it is essential to molecular booster networks involved in the promotion of the cardiac cell phenotype. The WNT/β-catenin signaling during embryonic development presents a biphasic role, being activated at early stages to induce the expression of mesendodermal markers such as Brachyury and Eomesodermin that must be inhibited at later stages to drive cardiac-lineage specification [140]. β-catenin stabilization and nuclear translocation are typically gained by adding CHIR99021, a potent inhibitor of GSK3β to the TFs or miRs cocktail. 

Originally, compounds were added to TFs or miRs cocktails to cooperatively enhance the effectiveness of the DCR protocol. Nevertheless, recent studies in mice and humans revealed the possibility of replacing the overexpression of exogenous genes and non-coding RNAs with a mixture of chemical compounds capable of inducing DCR [126,128,141]. Along with modulators of fibroblasts/cardiomyocytes gene signatures, a solo chemical cocktail requires the addition of epigenetic modulators, typically histone de-acetylase (HDAC) inhibitors and/or DNA/Histone methyltransferase inhibitors, to overcome the epigenetic barrier between different cell types. 

In 2015, Fu et al. achieved the first full chemical-induced DCR (CiDCR) of fibroblast into iCM with the cocktail CRFVPT (C-CHIR99021; R-RepSox; F-Forskolin; V-Valproic Acid; P-Parnate; T-TTNPB) in mice [141]. They optimized a two-stage protocol, in which the cocktail CRFVPT was used to initiate the induction process, then it was replaced by a cardiomyocytes-maintaining medium containing CHIR99021, PD0325901 (MEK/ERK inhibitor), Leukemia Inhibitory Factor (LIF), and insulin. Interestingly, this chemical-induced DCR seemed to pass through a cardiac progenitor stage [141]. Mechanistic understanding of how these diverse compounds specifically influence the cardiac reprogramming process remains limited, but Valproic Acid (VPA), a class I histone deacetylase inhibitor, together with Parnate (Lysine specific demethylase 1 inhibitor) are the epigenetic modulators that should be required to break through the epigenetic obstacles existing in fibroblasts, whereas Forskolin (a cAMP stimulator) and TTNPB (a synthetic retinoic acid analog) somehow should induce the characteristics of the designated cells.

Shortly after, a similar CiDCR protocol based on a combination of nine compounds in part overlapping the cocktail used for mouse cells [126] was sufficient and necessary to efficiently induce DCR of human fibroblasts, which could be transplanted into infarcted mouse hearts and converted efficiently into cardiomyocyte-like cells [126]. 

### 4.3. Targeting Cardiac Fibroblasts for In Vivo Direct Cardiac Reprogramming

The vast pool of CFs could serve as an endogenous source of new cardiomyocytes for regenerative therapy. In 2012, Qian et al. revealed that the injection of a retrovirus (ReV) encoding individual GMT factors in peri-infarcted areas of mouse hearts was able to reprogram resident CFs into iCMs [142]. By using lineage-tracing reporter mice, they excluded that iCMs were formed by fusion of endogenous CMs. Moreover, to induce CFs proliferation and improve reprogramming efficiency, they injected thymosin β4, a fibroblast-activating peptide, in combination with GMT factors and detected an increase in mature CM features according to structure, electrophysiology, and contractility. All these produced an improvement in heart structure and function 8 to 12 weeks post-injection [142].

Concurrently with Qian et al. [142], Song et al. reported that the injection of GHMT factors, encoded individually by ReV vectors, could trigger the conversion of endogenous cardiac fibroblasts into iCMs in a mouse model of MI [105]. The GHMT-transduced hearts showed a reduction of scar size and improved heart function 12 weeks post-infarction.

Since the use of multiple viral vectors encoding individual factors could decrease the reprogramming efficiency, several groups have focused on the use of polycistronic vectors encoding different splicing orders of GMT factors into infarcted mouse hearts, like the injection of a 2A-polycistronic ReV vector encoding TMG factors, which induced a twofold increase in the number of mature iCMs compared to the three single vectors [143], injection of PT2A-polycistronic ReV MGT vector, which increased the number of iCMs generated, but not their maturity [144], or the use of adenoviral vectors (AdV) encoding VEGF, to enhance vascularization, which was followed three weeks later by the injection of a TE2A-policystronic lentiviral (LeV) vectors encoding GTM factors in rat models of chronic MI [145]. Again, this showed an improvement in cardiac remodeling and function as well as a decrease in the number of myofibroblasts compared to monocystronic vectors [146].

As integrative viral vectors still present major concerns regarding the possible mutagenetic effects, some groups starting to use non-integrative viral vectors, such as Sendai virus (SeV) vectors. While AdV and LeV induced equivalent expression levels of GMT factors and had a similar transdifferentiation capacity compared to ReV polycistronic vectors [147], SeV polycistronic GMT vectors injected into mouse hearts after MI, achieved a greater efficiency of DCR with respect to ReVs [148] 1 week after injection, with a relative improvement in cardiac function and a reduction in fibrosis [148]. SeV-GMT generates iCMs through largely bona fide cardiac reprogramming and not through fusion events between cardiomyocytes and CFs [149]. Moreover, the beneficial effects of in vivo SeV-GMT reprogramming can be appreciated up to 12 weeks after MI in immunocompetent mice [149]. 

The improvement in several cardiac function parameters was also reported upon transduction of LeV vectors overexpressing miR combo to reprogram resident CFs into CMs in vivo in infarcted mouse hearts [113,150], as well as upon injection of GMT factors encoded in ReV vectors, in combination with repeated intraperitoneal administration of SB431542, a TGFβ inhibitor, and the WNT signaling inhibitor XAV939, in an MI mouse model [135].

Finally, the administration of CRFVPT cocktail with the addition of Rolipram, a selective phosphodiesterase-4 inhibitor, reduced fibrotic area and significantly improved heart functions in post-MI mouse models [151]. Nevertheless, the systemic administration of compounds induced significant weight loss indicating high toxicity grade [151].

Collectively, these studies not only demonstrated that DCR could be an effective strategy for cardiac regenerative interventions, but also indicated that the heart microenvironment can be more favorable to cardiac reprogramming than the in vitro culture conditions in terms of the efficiency and maturation of mouse iCMs [105,142,144].

Indeed, the local environment could have important influence on the efficiency of fibroblast direct reprogramming to cardiomyocytes [152]. One of the most intriguing aspects of in vivo reprogramming is that, irrespective of delivery vector or reprogramming cocktail, cardiac injury and the consequent myofibroblast activation are essential to achieve the meaningful generation of iCMs [105,142,150]. On the contrary, proliferation has been shown to be detrimental to cardiac reprogramming in vitro [123,153], as cells on a trajectory associated with successful reprogramming exit the cell cycle early in the process. 

Other aspects of the post-infarct microenvironment in the heart that could influence the reprogramming of activated fibroblasts are pro-inflammatory and pro-fibrotic cytokines released following the myocardial injury [5] to recruit macrophages and fibroblasts to the wounded area, in order to promote scar formation. Several groups have implicated pro-inflammatory responses in fibroblasts as critical influencers of reprogramming efficacy. The transcription factor Zinc Finger protein 281 (ZNF281) enhances iCM generation from mouse CFs by associating with the Nucleosome Remodeling and Deacetylase (NuRD) complex to repress pro-inflammatory gene expression [154], whereas the addition of anti-inflammatory small molecules like diclofenac can support reprogramming success in vitro [139], suggesting that pro-inflammatory factors produced by fibroblasts can sabotage the conversion to iCMs. In contrast, inflammation and immune responses are required for cardiac reprogramming in human fibroblasts in vitro [111,153]. The reason for this apparent discrepancy between mouse and human reprogramming needs to be clarified for future in vivo clinical application.

As previously mentioned, the best-known barrier to DCR are pro-fibrotic signals, whose inhibition promoted CFs reprogramming not only in vitro but also in vivo [135], as demonstrated by intraperitoneal administration of TGF-β and WNT inhibitors which, in addition to ReV GMT, increased the reprogramming efficiency and cardiac function in vivo [135]. Nevertheless, a recent single-cell RNA-seq analysis demonstrated that CFs in vivo are much more heterogeneous than previously thought [155,156], and the identification of the best CF subtype to target for in vivo cardiac reprogramming remains a key challenge.

Finally, mechanical properties of the damaged myocardium could also contribute to iCM reprogramming, as scar area is stiffer than the healthy myocardium due to ECM accumulation and fibrosis, and cells modify their gene expression pattern depending on the rigidity of the underlying matrix. This process was guided by two transcriptional co-activators, the Yes-Associated Protein 1 (YAP) and WW Domain Containing Transcription Regulator 1 (TAZ) [157], which might be other targets to improve in vivo cardiac reprogramming [158]. As a soft matrix, comparable to the native myocardium, promotes in vitro DCR via the inhibition of integrin, Rho/ROCK, actomyosin, and YAP/TAZ signaling [159], it is possible that the rigid scar tissue after MI due to YAP/TAZ activation might be an obstacle to CFs reprogramming.

## 5. Nanotechnology-Based Approaches for Direct Cardiac Reprogramming 

**Figure 3:**
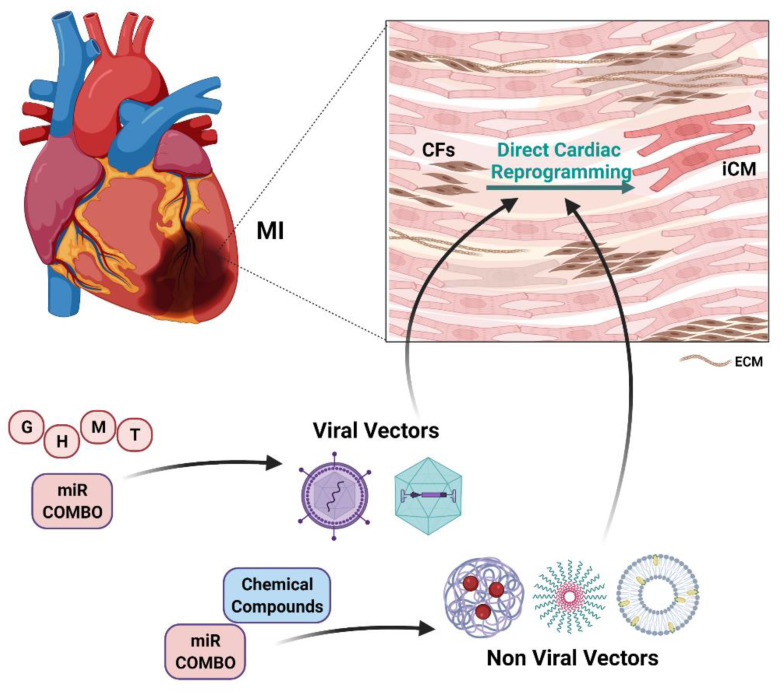
Strategies for in vivo direct cardiac reprogramming. Myocardial Infarction (MI); Cardiac Fibroblasts (CFs); induced Cardiomyocytes (iCMs); Extracellular Matrix (ECM); miR-1, miR-208, miR-133, miR499 (miR Combo); Gata4 (G); Hand2 (H); Mef2c (M); Tbx5 (T). Solid arrows indicate direct involvement. The figure is created with BioRender.

The possibility to regenerate the heart through DCR represents a fascinating as well as promising perspective. As highlighted, a great deal of effort has been devoted to the dissection of the DCR process to set an effective protocol based on the use of different agents (TFs, miRs, chemical compounds cocktails) [16]. Given the cellular targeting of these approaches, in the last decade interest has grown in the field of cellular delivery based on the use of nanotechnology-based approaches [7]. The use of nanotechnologies could help improving reprogramming efficiency and its in vivo feasibility, overcoming issues that have limited the delivery and the activity of reprogramming agents (Figure 3).

In general, nanotechnology-based systems could more efficiently deliver, in a target-specific way, reprogramming agents with high molecular weight such as nucleic acids or allow the simultaneous delivery of compounds with different physical and chemical properties. In addition, the desired reprogramming agents could be delivered to the identified cellular and tissue targets adjusting chemical, spatial and temporal variables, which have been shown to be significantly involved in the reprogramming efficiency. Finally, the targeted delivery of the reprogramming agents could significantly reduce cellular and systemic toxicity of the potentially harmful ones, facilitating its transition to a clinical setting [7]. Indeed, it has been already highlighted that the effectiveness of the reprogramming process could ultimately depend on the delivery of the reprogramming factors or compounds at the appropriate dose [110], and that the use of safe and specifically targeted delivery systems could allow the use of the lowest possible effective dose and its multiple administration, if needed.

The first response to concerns related to the use of potentially harmful genome-integrating viral vectors to deliver the reprogramming transcription factors led to the development of nonviral delivery systems such as nanoparticles. Even if not in the DCR setting, the use of a nanoparticle-based delivery system for the iPSC reprogramming factors led to higher transcription rates and, more interestingly, could be serially repeated to optimize reprogramming efficiency [160].

Similarly, to overcome the challenges related to mRNA transfection to reprogram mouse CFs to iCMs, an mRNA transfection system obtained by fusing polyarginine with the lipofectamine complex was used. This system led to efficient transfection with low toxicity, which allowed for multiple transfections of the GMT mRNAs over a two-week period. Interestingly, such iCMs showed increased expression of cardiomyocyte marker genes [161]. 

Recently, the cardiac reprogramming factors were successfully delivered using cationic gold nanoparticles loaded with GMT in both human and mouse somatic cells. This approach generated functional iCMs from MEFs in vitro and from CFs in vivo in mouse models of MI, resulting in low cytotoxicity and efficient in vivo DCR with a reduced infarct size and improved cardiac function [162]. 

Very recently, a miR combo cargo was loaded in polyethyleneimine coated nitrogen-enriched carbon dots for cardiac reprogramming. This approach also led to negligible toxicity and efficient DCR of CFs to iCMs with functional recovery in mouse infarcted hearts, without any genomic integration [163]. 

A very interesting approach led to the development of a non-viral biomimetic system obtained by coating FH peptide-modified neutrophil-mimicking membranes on silicon nanoparticles, which were loaded with miR Combo. Such nanoparticles used the natural inflammation-homing ability of neutrophil membrane protein and FH peptide’s high affinity to tenascin-C (TN-C) produced by CFs, to sequentially target CFs in the injured heart and deliver their cargo leading to efficient reprogramming and in improved cardiac function [164].

Concerning the targeted delivery of chemical compounds, two interesting experiences have been performed on nanotechnology-based approach. One of these approaches was conceived to deliver Forskolin and Repsox, which have previously been used in a reprogramming cocktail [128,141], using microporous annealing particles incapsulated into hydrogel blocks to drive tissue growth and local compound release. Although not aimed at cardiac reprogramming, these experiments showed that chemical reprogramming compounds could be released in a time and space dependent manner, retaining their ability to functionally modulate the activity of CMs, CFs, and ECs both in vitro and in vivo settings [165]. The second experience reports a nanoparticle based simultaneous delivery of CHIR99021 and Fibroblast Growth Factor 1 (FGF1). Even these two agents were effectively used in DCR experiments and were expected to synergistically enhance CMs cell *cycle* in vitro. Poly-lactic-co-glycolic acid nanoparticles were engineered to effectively release their cargo up to 4 weeks. The intramyocardial injection in mouse or pig models of MI of such nanocarriers was able to decrease cardiomyocyte apoptosis and increase angiogenesis thus reducing the infarct size and left ventricular remodeling, preserving cardiac function [166].

Very recently, an interesting proof-of principle experience encompassing many of the advantages of a nanotechnology-based approach to DCR has been performed. A small molecule inhibitor of GATA4-NKX2-5 loaded polymeric biocompatible elastomer, poly (glycerol sebacate) (PGS), coupled with collagen type I, was used to engineer a patch to be applied to the infarcted myocardium. Moreover, in the patch, a chemical agent was incorporated to improve cell conductivity and facilitate cell signaling. Even if in vitro experiments, this approach led to myoblasts attachment and proliferation to the patch meeting the mechanical, conductive, and biological needs necessary for a cardiac regenerative therapy [167]. 

## 6. Conclusions 

In the last decades, the evolution of new strategies to regenerate the injured myocardium based on cellular reprogramming represented a revolutionary new paradigm, providing a unique and efficient way to generate cell types of interest for cardiac repair by intervening on the plasticity of cell fate [16]. While the indirect reprogramming routes require an in vitro engineered 3D tissue to be transplant in vivo [80], the direct reprogramming would allow the administration of reprogramming factors directly in situ, thus holding great potential as a treatment for in vivo applications [16]. Nevertheless, significant challenges must be overcome before these strategies can be translated into novel therapies for human heart regeneration. 

A major obstacle for DCR is the low conversion efficiency and more efforts are necessary to gain insight on molecular mechanisms governing this process, not only to accelerate the optimization of reprogramming for upcoming clinical application but also to better understand the biology of fibroblast plasticity, in order to identify new potential reprogramming factors. 

The targeted delivery of reprogramming factors is also a major issue. The use of LeV or ReV delivery of genetic factors that induce fibroblast reprogramming has been previously linked to carcinogenesis [168] and the development of immune reactions in human patients [169]. AdV delivery, on the contrary, has been used in hundreds of clinical trials with no evidence of tumor formation in long-term follow up [170], but with a certain grade of heterogenic tropism, as AdVs often infect not only the heart but also other organs including the brain, lung, liver, and skeletal muscle [171]. Moreover, long-term expression of target genes has been linked to sudden death in pig studies [172]. As such, novel methods allowing the temporal over-expression of reprogramming factors would be of great importance. Preliminary results were already obtained by transient transfection of human CFs by non-viral vectors with miR combo [173].

The precise cardiac targeting for the delivery of therapeutics may be achieved mainly through nanotechnology-based systems. Nanocarriers would allow the delivery of reprogramming cargoes to the tissue targets adjusting chemical, spatial and temporal variables to reduce possible cellular and systemic toxicity. However, new targeting methods must be further explored to generate innovative delivery strategies to overcome the low targeting capability and treatment efficacy of current ones.

3D cardiac patches are a promising method in cardiac repair and can be either cellular or non-cellular. Cellular patches are generated through the seeding of different live cells into various 3D scaffolds and the future interdisciplinary cooperation between bioengineering and iPSC technology will be crucial in this research area. 

Compared with 3D cellular cardiac patches, non-cellular patches generated by seeding different cell derivatives into various 3D scaffolds, may have better stability, biocompatibility, modifiability, and low tumorigenicity and immunogenicity. 3D bioprinting technology has been widely utilized in cardiac repair by integrating biomaterials with different cell types to precisely pattern a cardiac structure [75]. However, this technology is still in the early stage and needs to be improved.

Finally, understanding the long-term consequences of novel cardiac regeneration strategies, particularly in large animal pre-clinical studies, is a mandatory step toward clinical translation. Overcoming these clinical barriers will undoubtedly begin a new era in the treatment of human heart disease.
